# Lipid dysregulation as a convergent pathway linking environmental exposures to stroke

**DOI:** 10.3389/fnagi.2026.1815998

**Published:** 2026-04-20

**Authors:** Maryline Santerre, Natalia Shcherbik, Bassel E. Sawaya

**Affiliations:** 1 FELS Cancer Institute for Personalized Medicine, Lewis Katz School of Medicine - Temple University, Philadelphia, PA, United States; 2 Department of Cell and Molecular Biology, School of Osteopathic Medicine, Rowan University, Stratford, NJ, United States; 3 Department of Cancer and Cellular Biology, Lewis Katz School of Medicine - Temple University, Philadelphia, PA, United States; 4 Department of Neural Sciences, Lewis Katz School of Medicine - Temple University, Philadelphia, PA, United States

**Keywords:** aging, environment, inflammation, lipids, stroke

## Abstract

Stroke remains the second leading cause of death globally, yet traditional risk factors explain only 50–60 percent of cases. Emerging evidence indicates that lipid dysregulation is a central mechanism linking environmental exposures to cerebrovascular vulnerability. Aging, chronic inflammation, infections, diet, inactivity, stress, sleep disorders, and toxins are associated with disruption of lipid homeostasis through oxidative stress-induced lipid peroxidation, cytokine-mediated metabolic reprogramming, blood-brain barrier disruption, ER stress-triggered lipid droplet formation, and mitochondrial dysfunction. These associations are supported by a combination of mechanistic, epidemiological, and clinical data, the strength of which varies across exposures and is explicitly evaluated throughout this review. Neuronal lipid droplets actively fuel synapses under stress, while membrane PUFA composition determines ischemic resilience. Lipid droplet accumulation, a hallmark of acute stroke, represents the potential endpoint of chronic environmental insults, creating metabolic fragility in which neurons may be less able to survive transient ischemia. Similar patterns in neurodegenerative disorders predict elevated stroke risk. However, direct causal evidence linking neuronal lipid droplet accumulation to stroke outcomes in humans remains limited, and this review explicitly distinguishes mechanistic hypotheses from clinically validated relationships. These factors are modifiable. Interventions targeting lipid homeostasis range from established therapies (statins, PPAR agonists, omega-3 fatty acids) to emerging approaches (mitochondria-ER stabilization, autophagy enhancement). This framework shifts stroke prevention from managing isolated risks to addressing the cumulative environmental burden on lipid metabolism, enabling precision prevention through lipidomic profiling and targeted intervention.

## Graphical Abstract

**Figure:**
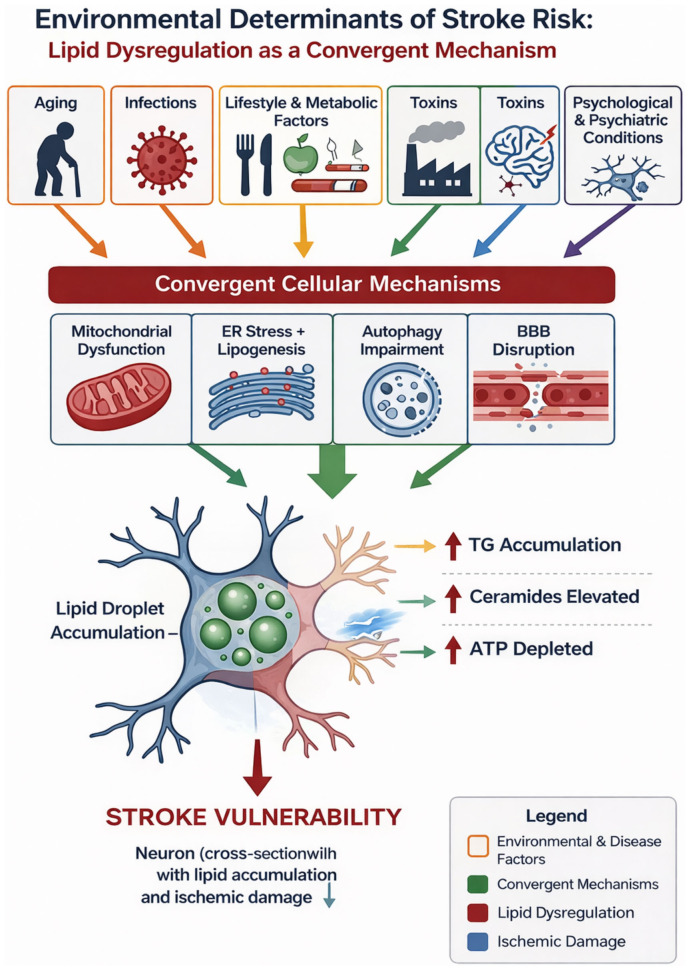
Environmental determinants of stroke risk and lipid dysregulation as a convergent mechanism for stroke vulnerability. This schematic illustrates environmental and disease-related factors that contribute to lipid dysregulation and increased stroke risk, including aging, infections, lifestyle and metabolic factors (e.g., diet, obesity, and tobacco use), environmental toxins, psychological and psychiatric conditions (including stress and PTSD), and neurodegenerative diseases. These factors converge on shared cellular mechanisms, including mitochondrial dysfunction, endoplasmic reticulum (ER) stress coupled with lipogenesis, autophagy impairment, and blood–brain barrier (BBB) disruption. These processes promote lipid imbalance characterized by lipid droplet and triglyceride (TG) accumulation, elevated ceramide levels, and ATP depletion, ultimately increasing neuronal vulnerability to ischemic stroke. The schematic uses icons and color-coded arrows for clarity: orange denotes environmental and systemic factors, green indicates convergent cellular mechanisms, red represents lipid dysregulation, and blue reflects ischemic neuronal damage.

## 1 Introduction

Stroke affects 15 million people annually and accounts for 5.5 million deaths worldwide (Feigin et al., 2025; GBD 2019 Stroke Collaborators, 2021). Traditional risk factors, including hypertension, diabetes, atrial fibrillation, dyslipidemia, and smoking, explain only 50–60 percent of cases (GBD 2021 Stroke Risk Factor Collaborators, 2024; O’Donnell et al., 2016; Soto-Cámara et al., 2020). Environmental exposures are hypothesized to disrupt cellular lipid homeostasis, rendering neurons metabolically vulnerable to ischemic injury (Boehme et al., 2017; Wang Y. et al., 2025; Wang et al., 2026; Yang Q. et al., 2025). However, for most environmental factors discussed in this review, the evidence base is epidemiological or mechanistic rather than derived from interventional clinical trials, and this distinction is maintained throughout.

Lipid dysregulation manifests as neuronal lipid droplet accumulation, cytoplasmic organelles containing triglycerides and cholesterol esters that appear during severe metabolic stress (Singh et al., 2023; Zhang et al., 2025). Healthy neurons derive approximately 95% of ATP from glucose oxidation under normal conditions (Magistretti and Allaman, 2015), with minimal lipid stores; lipid droplet formation marks profound metabolic dysfunction (Ralhan et al., 2021; Zhong et al., 2025). These accumulations occur in both acute stroke and chronic neurodegenerative disorders, suggesting shared pathways (Fu et al., 2025). The lipid profile observed under ischemic conditions is relatively conserved, characterized by polyunsaturated triglycerides derived from membrane phospholipid degradation, plasmalogens, increased ceramides, and sphingolipid dysregulation (Kloska et al., 2020; Wang J. et al., 2025). Lipid alterations also impair the cerebrovascular endothelium, promoting barrier instability and prothrombotic states (Andreone et al., 2017; Iadecola, 2017; Kloska et al., 2020; Profaci et al., 2020; Sweeney et al., 2019).

Environmental factors may modulate stroke risk through diverse mechanisms that converge on shared pathways, including mitochondrial-associated ER membrane (MAM) dysfunction, ER stress-activated lipogenic signaling, reduced autophagy-mediated lipid degradation, and blood-brain barrier disruption (Khoshnam et al., 2017; Kuzu et al., 2025). This convergence hypothesis, while mechanistically plausible, is largely based on experimental models; human longitudinal data directly linking cumulative environmental lipid burden to stroke incidence are limited and represent a key gap addressed in the knowledge gaps section.

## 2 Cellular mechanisms: how environment disrupts lipid homeostasis

### 2.1 Mitochondrial-ER contact sites: the lipid synthesis hub

MAMs are 10–30 nm contact sites where ER and mitochondrial membranes are tethered (Stoica et al., 2014; Truong et al., 2025; Vance, 2020), concentrating enzymes for phospholipid biosynthesis (Kuzu et al., 2025; van Vliet and Agostinis, 2018; Vance, 2015) and regulating calcium transfer, autophagosome formation, and inflammasome assembly (Giacomello et al., 2020). Environmental insults have been associated with disruption of MAM integrity through several mechanisms: oxidative stress from tobacco smoke, pollution, or inflammation may oxidize VAPB cysteine residues, potentially impairing PTPIP51 binding (Arjona et al., 2023; Hetz et al., 2020; Nguyen et al., 2023). Direct evidence for oxidative modification of VAPB in human stroke tissue is currently lacking; this mechanism is inferred from *in vitro* and animal studies (Arjona et al., 2023). Viral proteins, including HIV Tat and SARS-CoV-2 ORF3a, have been shown in experimental models to disrupt MAM contacts (Arjona et al., 2023; Lee et al., 2021). Advanced glycation end-products generated during high-temperature cooking have been proposed to cross-link MAM proteins, thereby reducing contact-site dynamics, though direct evidence in neural tissue is limited (Markovinovic et al., 2024; Müller et al., 2018). Aging reduces VAPB-PTPIP51 complexes (Miller and Gomez-Suaga, 2023; Stoica et al., 2014), impairing phospholipid synthesis and calcium buffering. MAM disruption reduces the conversion of phosphatidylserine to phosphatidylethanolamine, which in experimental models forces cells to compensate by synthesizing triglycerides and forming lipid droplets (Arbaizar-Rovirosa et al., 2023; Bui et al., 2026; Wei et al., 2024; Yang et al., 2020; Yang J. et al., 2025).

Recent studies underscore the importance of neuronal lipid metabolism under neurological stress. Presynaptic lipid droplet triglycerides serve as rapidly mobilizable fuel for mitochondrial energy during periods of high neuronal demand or glucose limitation, and inhibiting triglyceride hydrolysis or mitochondrial fatty acid transport impairs synaptic function and thermoregulation in mice (Kumar et al., 2025). Additionally, neuronal PUFA depletion has been associated with neurodegeneration, and restoring fatty acid desaturation improves phenotypes in patient-derived neurons (Giblin et al., 2025), suggesting that maintaining lipid balance may be critical for neuronal resilience and represents a potential (Dakal et al., 2025), though not yet clinically validated.

### 2.2 ER stress and lipogenic signaling

The unfolded protein response is activated by ER stress (Bhattarai et al., 2021; Walter and Ron, 2011). While acute UPR is adaptive, chronic activation due to persistent inflammation, viral infection, or metabolic overload has been shown in experimental models to induce lipogenesis (Hetz et al., 2020; Walter and Ron, 2011). PERK phosphorylates eIF2alpha, inducing ATF4 and CHOP, which upregulate SREBP-1c, the master lipogenic transcription factor (Borradaile et al., 2006). SREBP-1c increases fatty acid synthase, acetyl-CoA carboxylase, and stearoyl-CoA desaturase, driving *de novo* lipogenesis (Lee et al., 2008). IRE1alpha splices XBP1, activating genes for phospholipid synthesis and ER expansion (Lee et al., 2008). Prolonged UPR overwhelms ER capacity, channeling excess lipids into cytoplasmic droplets in experimental models (Volmer et al., 2013). Whether this sequence operates identically in human neurons under environmental stress conditions has not been directly demonstrated and represents an important knowledge gap. Neurons, optimized for glucose metabolism, are poorly tolerant of ER stress; lipid droplet accumulation in cell and animal models signals impending dysfunction.

### 2.3 Autophagy failure and lipid accumulation

Lipophagy, the selective autophagy of lipid droplets, maintains lipid homeostasis (Singh et al., 2009). Environmental stressors have been associated with impaired autophagy through several mechanisms: aging reduces autophagosome-lysosome fusion (Barbosa et al., 2019; Stavoe et al., 2019; Tsong et al., 2023); mTOR hyperactivation from insulin resistance and inflammation suppresses autophagy initiation (Betz and Hall, 2013); lysosomal dysfunction from heavy metal accumulation and oxidative damage prevents lipid catabolism (López-Otín et al., 2023). Viral proteins, including HIV Tat and HCV core, have been shown *in vitro* to inhibit autophagy by sequestering Beclin-1 (Mizushima and Levine, 2020). Impaired lipophagy in animal models causes lipid droplet accumulation, shifts neurons from glucose to fatty acid oxidation, reduces efficiency, and increases ROS generation, thereby exacerbating damage (Aman et al., 2021).

### 2.4 Blood-brain barrier disruption

The BBB maintains CNS lipid homeostasis by excluding atherogenic lipoproteins (Andreone et al., 2017). Environmental insults have been associated with breach of this barrier: chronic hypertension degrades endothelial tight junctions (Iadecola, 2017); systemic inflammation from infection and air pollution has been shown in experimental models to induce matrix metalloproteinase-mediated degradation of the basement membrane (Profaci et al., 2020; Sweeney et al., 2019); and oxidized LDL from smoking or diet directly damages endothelial cells *in vitro* (Andreone et al., 2017). Evidence that these mechanisms collectively produce clinically relevant BBB disruption in stroke-prone humans is largely inferential; direct human data are discussed in section 7. BBB breakdown allows peripheral lipoprotein influx, overwhelming neuronal lipid handling. Extravasated lipoproteins may activate microglia, amplify neuroinflammation, and further impair barrier function, creating a self-amplifying cycle (Nation et al., 2019; Sweeney et al., 2019).

As summarized in Table 1, diverse exposures, including aging, metabolic disorders, lifestyle factors, and environmental toxins, have been associated with disruption of lipid homeostasis through shared mechanisms, including mitochondrial dysfunction, ER stress, impaired autophagy, and BBB breakdown. The evidence level for each exposure varies and is indicated in Table 1. Accumulation of multiple exposures likely produces additive or potentially synergistic disruption of lipid metabolism, contributing to the observed increase in stroke risk with cumulative environmental burden (O’Donnell et al., 2016; Wang Y. et al., 2025).

**TABLE 1:** Environmental factors, mechanisms, and lipid-mediated stroke risk.

Environmental factor	Primary mechanism	Lipid pathways affected	Evidence level	References
Aging	Decline in autophagy and mitochondrial function	Lipid droplet accumulation, phospholipid oxidation, increased ceramide production	Strong (human and animal studies)	(Biondi-Zoccai et al., 2019; McEvoy et al., 2015; Pan et al., 2019; Valavanidis et al., 2009; Walter and Ron, 2011; Yan et al., 2021)
Hypertension	Endothelial shear stress and oxidative stress	Membrane phospholipid peroxidation, sphingolipid dysregulation	Strong (clinical studies)	(Khoshnam et al., 2017; Stoica et al., 2014; Wang et al., 2026)
Type 2 diabetes	Insulin resistance and chronic hyperglycemia	Triglyceride accumulation, increased ceramide synthesis, PUFA oxidation	Strong (clinical studies)	(McEvoy et al., 2015; Wang et al., 2026; Yan et al., 2021)
Cigarette smoking	Oxidative stress and inflammatory activation	Lipid peroxidation, oxidized LDL formation, HDL dysfunction	Strong (clinical studies)	(O’Donnell et al., 2016; Wang et al., 2026)
Air pollution (PM2.5)	Particulate-induced systemic inflammation and oxidative stress	Membrane lipid oxidation, systemic dyslipidemia	Moderate to strong (epidemiological studies)	(Boehme et al., 2017; Wang et al., 2026)
Viral infections (e.g., influenza, COVID-19)	Acute inflammatory responses and endothelial activation	Phospholipase activation, membrane lipid remodeling	Moderate (clinical studies)	(Hetz et al., 2020; Moldogazieva et al., 2019; Nguyen et al., 2023)
Bacterial infections (e.g., periodontitis, *H. pylori*)	Chronic low-grade systemic inflammation	LDL oxidation, HDL dysfunction	Moderate (observational studies)	(O’Donnell et al., 2016; Wang et al., 2026)
Western diet	High intake of saturated fats and refined carbohydrates	↑ Triglycerides, elevated LDL, reduced membrane fluidity	Strong (clinical trials and population studies)	(Boehme et al., 2017; Lee et al., 2008; O’Donnell et al., 2016; Rambold et al., 2015)
Mediterranean diet	Enrichment in MUFAs, PUFAs, and antioxidants	↑ HDL, reduced inflammatory lipid mediators, improved membrane fluidity	Strong (PREDIMED and related trials)	(O’Donnell et al., 2016; Wang et al., 2026)
Physical inactivity	Metabolic dysfunction and increased adiposity	Visceral fat accumulation, ectopic lipid deposition	Strong (observational and clinical studies)	(O’Donnell et al., 2016; Wang et al., 2026)
Chronic psychological stress	HPA axis activation and elevated cortisol levels	Visceral adiposity, lipoprotein imbalance	Moderate (mechanistic and epidemiological studies)	(Boehme et al., 2017; Wang et al., 2026)
Sleep deprivation	Metabolic dysregulation and systemic inflammation	↑ triglycerides, impaired insulin sensitivity	Moderate (experimental and clinical studies)	(Boehme et al., 2017; Wang et al., 2026)
Obesity	Adipose tissue inflammation and insulin resistance	↑ free fatty acids, increased ceramide production, ectopic lipid deposition	Strong (clinical and mechanistic studies)	(McEvoy et al., 2015; O’Donnell et al., 2016; Wang et al., 2026; Yan et al., 2021)
Alcohol (Heavy Consumption)	Hepatic dysfunction and oxidative stress	↑ Triglycerides, variable HDL changes, membrane lipid disruption	Moderate to strong (clinical studies)	(O’Donnell et al., 2016; Wang et al., 2026)
Alcohol (Moderate Consumption)	Potential lipoprotein modulation	↑ HDL levels (controversial protective effect)	Weak to moderate (conflicting evidence)	(O’Donnell et al., 2016; Wang et al., 2026)

Environmental exposures converge on shared lipid metabolic pathways that influence stroke susceptibility. This table summarizes major environmental factors (aging, lifestyle, infections, toxins), their primary cellular mechanisms that disrupt lipid homeostasis, affected lipid pathways, the strength of epidemiological evidence, and representative references. Evidence levels: Strong = consistent findings from randomized trials or large prospective cohorts; Moderate = observational data with mechanistic support; Weak = limited or inconsistent evidence. LD, lipid droplet; ROS, reactive oxygen species; PUFA, polyunsaturated fatty acids; MUFA, monounsaturated fatty acids; oxLDL, oxidized low-density lipoprotein; HDL, high-density lipoprotein; LDL, low-density lipoprotein; HPA, hypothalamic-pituitary-adrenal; PM2.5, particulate matter ≤ 2.5 μm.

**KEY CONCEPT: Lipid Dysregulation as a Convergent Mechanism**

*The following mechanisms represent a conceptual framework supported by varying degrees of experimental, epidemiological, and clinical evidence. Each is marked accordingly in the text: Multiple environmental exposures converge on lipid metabolic pathways through shared cellular mechanisms* (Dakal et al., 2025).

- *Mitochondrial dysfunction reduces the capacity for fatty acid beta-oxidation, promoting lipid accumulation* (Giacomello et al., 2020; Nguyen et al., 2023).
- *ER stress activates lipogenic transcription factors SREBP and ChREBP, increasing de novo lipid synthesis* (Borradaile et al., 2006; Hetz et al., 2020; Lee et al., 2008).
- *Autophagy impairment prevents lipophagy-mediated clearance of lipid droplets and damaged organelles* (Aman et al., 2021; Betz and Hall, 2013; López-Otín et al., 2023; Mizushima and Levine, 2020; Singh et al., 2009).
- *BBB breakdown permits peripheral lipid influx and disrupts CNS lipid homeostasis* (Andreone et al., 2017; Iadecola, 2017; Nation et al., 2019; Profaci et al., 2020; Sweeney et al., 2019).

*This convergence model is mechanistically plausible and consistent with epidemiological data showing supra-additive stroke risk with multiple risk factors* (O’Donnell et al., 2016; Wang Y. et al., 2025) *but has not been tested prospectively as an integrated lipid-centric framework in humans.*

## 3 Aging: the dominant environmental factor

Aging is the strongest non-modifiable stroke risk factor, with incidence increasing exponentially after age 55 (Benjamin et al., 2019; Feigin et al., 2009). While aging is inevitable, the rate of biological aging varies with environmental exposures. Age-related lipid dysregulation involves multiple converging mechanisms: mitochondrial Complex I and IV activity declines with aging (Pollard et al., 2016), impairing fatty acid oxidation and promoting lipid accumulation (López-Otín et al., 2023); membrane PUFA content decreases with aging (Weiser et al., 2016), increasing rigidity and ischemic vulnerability (Arbaizar-Rovirosa et al., 2023; Wei et al., 2024; Yang et al., 2020; Yang J. et al., 2025); autophagy declines with aging (Cheon et al., 2019) preventing lipid clearance (López-Otín et al., 2023); MAM integrity degrades with aging, disrupting phospholipid synthesis; and chronic low-grade inflammation activates lipogenic pathways (Di Micco et al., 2021; Ferrucci and Fabbri, 2018; Khosla et al., 2020). Aged neurons in experimental models exhibit constitutive lipid droplets even in the absence of acute stress, creating baseline vulnerability (Arbaizar-Rovirosa et al., 2023; Wei et al., 2024; Yang J. et al., 2025).

Whether this baseline accumulation of lipid droplets directly increases ischemic susceptibility in aging humans remains to be established in prospective studies. Interventions targeting these pathways, including autophagy enhancers, mitochondrial support, and anti-inflammatory strategies, have shown promise in animal models and require validation in human stroke prevention trials (Di Micco et al., 2021; Khosla et al., 2020).

Critically, lipid accumulation patterns in aged brains share features with those in stroke: polyunsaturated triglycerides dominate, plasmalogens decrease (Bozelli et al., 2021), and ceramides increase (Arbaizar-Rovirosa et al., 2023; Filippov et al., 2012; Wei et al., 2024; Yang et al., 2020; Yang J. et al., 2025). Aged brains exhibit ATP depletion, NAD decline, and metabolic signatures that overlap with those of the ischemic penumbra, suggesting, though not proving, that aging creates a chronic metabolic vulnerability. The concept of biological age versus chronological age has therapeutic implications. Interventions that slow biological aging, including caloric restriction, rapamycin, NAD+ precursors, and senolytics, improve lipid metabolism and reduce stroke risk in animal models (Di Micco et al., 2021; Khosla et al., 2020), though human clinical trial evidence for stroke-specific outcomes remains limited for most of these agents. Human studies of centenarians reveal preserved lipid homeostasis with lower triglycerides, higher HDL, and reduced oxidized lipids (Khosla et al., 2020; López-Otín et al., 2023), demonstrating that age-related lipid dysfunction is not universal and may be modifiable.

## 4 Infections: acute and chronic triggers

### 4.1 Viral infections and stroke risk

Influenza, COVID-19, HIV, and HCV have been associated with increased stroke risk through mechanisms involving lipid disruption (Madjid et al., 2020; Udell et al., 2013; Warren-Gash et al., 2018). Acute viral infections, including influenza and SARS-CoV-2, trigger systemic inflammation, which has been shown to activate SREBP-1c and drive hepatic lipogenesis; cytokine storm has been associated with impaired lipoprotein metabolism, elevating triglycerides (Madjid et al., 2020; Saballs et al., 2024). Viral proteins have been shown in experimental models to directly disrupt cellular lipids: HIV Tat induces neuronal lipid droplets via ER stress (Arjona et al., 2023); SARS-CoV-2 ORF3a has been proposed to disrupt MAMs, impairing phospholipid synthesis (Lee et al., 2021). Direct evidence for SARS-CoV-2 ORF3a-mediated MAM disruption in human neural tissue is limited; this is inferred from experimental models. Chronic viral infections maintain persistent inflammation and metabolic reprogramming. HIV-associated neurocognitive disorders involve lipid droplet accumulation in neurons and microglia, and people living with HIV have increased stroke risk (Benjamin et al., 2012; Harding et al., 2021). HCV has been associated with hepatic steatosis and systemic dyslipidemia, correlating with stroke incidence (Elgretli et al., 2023).

### 4.2 Bacterial infections and vascular inflammation

Periodontal disease, H. pylori, and Chlamydia pneumoniae have been epidemiologically associated with stroke (Leira et al., 2019; Winning et al., 2017). Chronic bacterial infections are associated with sustained elevations of IL-6, TNF-alpha, and CRP, which in experimental models activate hepatic lipogenesis and impair reverse cholesterol transport (Leira et al., 2019). Bacterial endotoxin LPS has been shown in animal models to disrupt the BBB via TLR4 signaling, increasing permeability and allowing lipoprotein influx. Molecular mimicry has been hypothesized to induce autoantibodies against oxidized LDL, promoting atherogenesis (Hayashi et al., 2010). Causal inference from these associations is limited by confounding in observational studies; randomized trials of periodontal treatment have not consistently reduced stroke incidence (Sen et al., 2023). Periodontal pathogens have been detected in atherosclerotic plaques, suggesting local inflammatory mechanisms (Hayashi et al., 2010).

## 5 Lifestyle factors: modifiable environmental risks

### 5.1 Tobacco: the archetypal environmental toxin

Smoking increases stroke risk 2–4-fold (Pan et al., 2019), through mechanisms involving direct lipid toxicity: nicotine has been shown to activate sympathetic signaling, inducing lipolysis and elevating circulating free fatty acids (McEvoy et al., 2015); oxidants oxidize LDL, creating atherogenic particles that are preferentially taken up by macrophages (Leira et al., 2019; Valavanidis et al., 2009); carbon monoxide impairs mitochondrial respiration, which in experimental models shifts metabolism toward lipogenesis. Smoking has been associated with reduced HDL (Maeda et al., 2003) and increased triglycerides (van der Plas et al., 2023). Cessation progressively reduces risk; after 5 years, risk approaches that of non-smokers (Moldogazieva et al., 2019; Pan et al., 2019).

### 5.2 Diet: nutritional determinants of lipid homeostasis

High saturated fat intake above 10 percent of calories has been associated with increased stroke risk (Siri-Tarino et al., 2010), elevating LDL and promoting atherogenesis. Trans fats have been shown to raise LDL while lowering HDL and have been associated with substantially elevated risk of cerebrovascular disease (de Souza et al., 2015). Excess refined carbohydrates are associated with postprandial hyperglycemia and insulin resistance, which activate SREBP-1c. Conversely, Mediterranean diet patterns have been associated with reduced stroke risk in the PREDIMED trial (Estruch et al., 2018), through PUFA incorporation, anti-inflammatory polyphenols, and improved lipoprotein profiles (Meslier et al., 2020). Omega-3s EPA and DHA reduce triglycerides, inhibit VLDL synthesis, and incorporate into membranes, with evidence from randomized trials showing cardiovascular benefit in high-risk populations (Hu et al., 2019), though stroke-specific outcomes vary across trials (Hu et al., 2019; Yang et al., 2020).

### 5.3 Physical activity: exercise and metabolic resilience

Sedentary behavior has been associated with increased stroke risk (Lee et al., 2012). Exercise has been shown to activate AMPK, stimulating fatty acid oxidation and autophagy (Aman et al., 2021; Giacomello et al., 2020); increase lipoprotein lipase, enhancing VLDL clearance (Wahid et al., 2016); upregulate ABCA1, promoting reverse cholesterol transport; and improve insulin sensitivity, reducing lipogenic drive. Moderate activity of 150 min per week has been associated with reduced stroke risk in prospective cohort studies (Lee et al., 2012; Wahid et al., 2016). Randomized trial evidence specifically testing exercise for stroke prevention through lipid mechanisms is limited; most evidence derives from observational studies (Lee et al., 2012; Wahid et al., 2016).

### 5.4 Sleep: circadian control of lipid metabolism

Sleep deprivation below 6 h per night has been associated with increased stroke risk (Jike et al., 2018). Insufficient sleep has been associated with elevated cortisol levels, which induce lipolysis (McEwen, 2017); impaired insulin sensitivity, which activates lipogenesis; and reduced glymphatic clearance, which impairs brain lipid efflux (Dyar et al., 2018). Sleep apnea produces intermittent hypoxia, generating ROS that have been shown to oxidize lipids and disrupt the BBB in experimental models (Lavie, 2015). Treatment with CPAP has been associated with reduced cardiovascular events in randomized trials (McEvoy et al., 2016), though the stroke-specific benefit through lipid mechanisms specifically has not been isolated.

### 5.5 Psychological stress: neuroendocrine regulation of lipids

Chronic psychological stress has been associated with increased stroke risk by 30–50 percent in epidemiological studies (Tenk et al., 2018), whereas acute severe stress has been associated with transient risk increase in case-control studies (Lindert et al., 2020). PTSD has been associated with elevated lifetime stroke risk in veteran and civilian cohorts (Nanavati et al., 2023). Stress activates hypothalamic-pituitary-adrenal signaling and chronically elevates cortisol, which in metabolic studies promotes lipolysis, hepatic VLDL production, and insulin resistance, thereby increasing circulating lipids (McEwen, 2017). Stress has also been associated with compromised blood-brain barrier integrity and enhanced oxidative stress through sympathetic activation (Kealy et al., 2020).

## 6 Environmental toxins: ubiquitous exposures

### 6.1 Air pollution: particulate matter and stroke

Particulate matter PM2.5 has been associated with increased stroke risk in epidemiological studies (Yang et al., 2018). Ultrafine particles smaller than 0.1 micrometers have been detected in human brain tissue and have been shown to cross the BBB directly (Maher et al., 2016). Mechanisms include oxidative stress generating lipid peroxidation products (Brook et al., 2010; Furlong and Klimentidis, 2020); systemic inflammation that activates lipogenic pathways (Brook et al., 2010); and endothelial dysfunction that impairs lipoprotein metabolism (Brook et al., 2010). Most mechanistic evidence derives from experimental models; direct evidence that PM2.5-induced lipid peroxidation drives stroke risk in humans requires further longitudinal investigation (Bourdrel et al., 2017). Traffic-related pollution has been associated with carotid intima-media thickness and plaque lipid content in epidemiological studies (Bourdrel et al., 2017).

### 6.2 Heavy metals: neurotoxic element exposure

Lead, mercury, and cadmium accumulate in lipid-rich tissues (GBD 2019 Stroke Collaborators, 2021). Lead has been shown to inhibit delta-aminolevulinic acid dehydratase, impairing heme synthesis and increasing oxidative stress (Lanphear et al., 2018; Nguyen et al., 2023); it displaces calcium from enzymes, thereby disrupting lipid metabolism in experimental models (Nguyen et al., 2023). Mercury binds selenoproteins, impairing antioxidant defense and allowing lipid peroxidation (Rasinger et al., 2017). Cadmium induces metallothionein, sequestering zinc and copper required for lipid-metabolizing enzymes (Tellez-Plaza et al., 2012). Blood lead above 5 micrograms per deciliter has been associated with increased stroke risk in epidemiological studies (Lanphear et al., 2018). The mechanistic link between heavy metal-induced lipid peroxidation and stroke has primarily been established in animal models; large-scale human mechanistic studies are limited.

### 6.3 Pesticides and industrial chemicals

Organophosphates and organochlorines accumulate in adipose tissue (Lee et al., 2011). Organophosphates have been shown to inhibit acetylcholinesterase, leading to neuronal calcium overload and activating lipases in experimental models (Mostafalou and Abdollahi, 2017); they also disrupt mitochondrial function, thereby impairing fatty acid oxidation (Hetz et al., 2020; Nguyen et al., 2023). Persistent organic pollutants act as endocrine disruptors, altering lipid metabolism by modulating nuclear receptors (Cheek et al., 1999). High POP levels have been associated with increased triglycerides and stroke risk in observational studies (Lee et al., 2011). Causal inference is limited by the observational nature of these data and potential confounding by socioeconomic and dietary factors.

## 7 Convergent mechanisms: the lipid-stroke axis

Despite diverse origins, environmental insults have been associated with shared lipid-disrupting pathways: oxidative stress generates lipid peroxides and in experimental models activates SREBP-1c (Moldogazieva et al., 2019; Nguyen et al., 2023); inflammation via IL-6 and TNF-alpha has been associated with hepatic lipogenesis and impaired reverse cholesterol transport in human studies (Ferrucci and Fabbri, 2018; Khovidhunkit et al., 2004); BBB disruption has been demonstrated in experimental and some human imaging studies (Nation et al., 2019; Profaci et al., 2020; Sweeney et al., 2019); ER stress activates UPR-driven lipogenesis in cell models (Bhattarai et al., 2021; Hetz et al., 2020); mitochondrial dysfunction impairs fatty acid oxidation in experimental models (Giacomello et al., 2020; Nguyen et al., 2023); and autophagy decline has been shown to prevent lipid clearance (Aman et al., 2021; Betz and Hall, 2013). These pathways may amplify each other: ROS can activate inflammatory signaling (Nguyen et al., 2023); inflammation can disrupt the BBB (Sweeney et al., 2019); BBB breakdown allows entry of oxidized LDL (Andreone et al., 2017); oxidized LDL generates more ROS (Moldogazieva et al., 2019; Nguyen et al., 2023). Whether these pathways interact synergistically *in vivo* in humans to produce supra-additive stroke risk beyond what can be attributed to individual risk factors has not been tested prospectively. The multi-hit scenario, in which cumulative environmental burden dramatically elevates stroke risk beyond the sum of individual contributions, is epidemiologically supported (Dakal et al., 2025; O’Donnell et al., 2016; Wang Y. et al., 2025) but mechanistically remains a plausible hypothesis that requires prospective lipidomic validation.

## 8 Therapeutic strategies: targeting lipid homeostasis

### 8.1 Lifestyle interventions: first-line prevention

The Mediterranean diet reduced stroke risk by approximately 30 percent in the PREDIMED randomized trial (Estruch et al., 2018; Meslier et al., 2020), through PUFA incorporation, anti-inflammatory effects, and improved lipid profiles. Exercise of 150 min per week at moderate intensity has been associated with lower stroke risk in prospective cohort studies (Lee et al., 2012; Wahid et al., 2016) through AMPK activation (Aman et al., 2021; Giacomello et al., 2020), enhanced lipoprotein metabolism, and stimulation of autophagy (Aman et al., 2021; López-Otín et al., 2023). Randomized trial evidence for exercise specifically targeting neuronal lipid homeostasis as a stroke prevention mechanism is not yet available. Smoking cessation yields a time-dependent benefit; 5 years post-cessation, risk approaches that of non-smokers (Pan et al., 2019). Sleep optimization, including CPAP for sleep apnea, has been shown to reduce cardiovascular events in the SAVE trial (McEvoy et al., 2016), though stroke-specific lipid-mediated benefit has not been isolated.

### 8.2 Pharmacologic lipid-modifying therapies

Statins reduce stroke risk by 20–25 percent in randomized trials (Andreone et al., 2017) through LDL-lowering and pleiotropic effects, including plaque stabilization and reduced inflammation. PCSK9 inhibitors provide an additional 15 percent relative risk reduction in high-risk patients (Jackler and Ramamurthi, 2019; Wang Y. et al., 2025). Fibrates lower triglycerides but have not demonstrated consistent stroke benefit in clinical trials (Kersten and Stienstra, 2017) and are currently not recommended for stroke prevention specifically. Omega-3 fatty acids at 4 grams per day, with EPA, reduced cardiovascular events by 25 percent in the REDUCE-IT trial in patients with elevated triglycerides (Hu et al., 2019); the stroke-specific benefit and the contribution of lipid versus anti-inflammatory mechanisms remain debated. PPAR-gamma agonists, including pioglitazone, reduced the risk of recurrent stroke in the IRIS trial among insulin-resistant patients (Forman et al., 2022; Sundararajan et al., 2005), providing randomized evidence for PPAR-targeted stroke prevention in this specific population. Omega-3 supplementation results vary across trials, with benefits primarily observed in populations with low baseline fish consumption (Hu et al., 2019; Yang et al., 2020). Clinicians should note that lipid-lowering therapy for stroke prevention is well supported for LDL reduction; the role of triglyceride-targeted or mechanism-based lipid therapies specifically requires further trial data. Table 2 details the current landscape of lipid-directed stroke prevention strategies, including their mechanisms, clinical evidence, and implementation status.

**TABLE 2:** Therapeutic interventions targeting lipid pathways in stroke prevention.

Intervention	Primary target	Mechanism	Clinical evidence	Current status	Key limitations	References
Statins	LDL, inflammation	HMG-CoA reductase inhibition	RRR 20–25%	Standard therapy	Myopathy risk	(Jackler and Ramamurthi, 2019; Mortensen and Nordestgaard, 2020; Wang et al., 2026)
High-intensity statins	Aggressive LDL ↓	Same mechanism	RRR 30–35%	Guideline recommended	↑ adverse effects	(Jackler and Ramamurthi, 2019; Kealy et al., 2020; Wang et al., 2026)
Ezetimibe	Cholesterol absorption	NPC1L1 inhibition	Added RRR 6–8%	Add-on therapy	Modest monotherapy efficacy	(Jackler and Ramamurthi, 2019; Wang et al., 2026)
PCSK9 inhibitors	LDL clearance	Monoclonal antibody	RRR 15–20%	High-risk patients	Cost, injections	(Jackler and Ramamurthi, 2019; Wang et al., 2026)
Inclisiran	PCSK9 synthesis	RNA interference	∼50% LDL reduction	Emerging therapy	Limited long-term data	(Wang et al., 2026; Yang Q. et al., 2025b)
Fibrates	Triglycerides	PPARα activation	No stroke reduction	Not recommended	Inconsistent benefit	(Khoshnam et al., 2017; Wang et al., 2026)
Omega-3 fatty acids	Membrane composition	Anti-inflammatory, TG reduction	Mixed results	Prescription EPA approved	Formulation variability	(Khoshnam et al., 2017; Lee et al., 2008; Rambold et al., 2015)
Mediterranean diet	Multiple pathways	PUFA/MUFA, antioxidants	RRR ∼30%	Strong recommendation	Behavioral adherence	(O’Donnell et al., 2016; Wang et al., 2026)
Aerobic exercise	HDL, visceral fat	Lipid oxidation, mitochondrial biogenesis	RRR 20–30%	Strong recommendation	Compliance variability	(McEvoy et al., 2015; O’Donnell et al., 2016; Wang et al., 2026; Yan et al., 2021)
Weight loss	Visceral adiposity	Improves insulin sensitivity	RRR 15–25%	Recommended	Weight regain	(McEvoy et al., 2015; O’Donnell et al., 2016; Wang et al., 2026; Yan et al., 2021)
Bempedoic acid	LDL	ATP citrate lyase inhibition	RRR ∼13%	New therapy	Limited long-term data	(Wang et al., 2026; Yang Q. et al., 2025b)
Lipid droplet modulators	LD dynamics	Regulate lipid mobilization	Preclinical only	Investigational	No human data	(Fu et al., 2025; Giacomello et al., 2020; Giblin et al., 2025; Ralhan et al., 2021; Walter and Ron, 2011; Zhong et al., 2025)
PUFA supplementation	Membrane phospholipids	DHA/EPA incorporation	Mixed results	Investigational	Dose uncertainty	(Khoshnam et al., 2017; Lee et al., 2008; Rambold et al., 2015)
Antioxidants	Lipid peroxidation	Free radical scavenging	No benefit	Not recommended	Failed RCTs	(Khoshnam et al., 2017; Kumar et al., 2025)
Niacin	HDL, triglycerides	Complex lipid modulation	No stroke benefit	Not recommended	Adverse effects	(Mortensen and Nordestgaard, 2020; Wang et al., 2026)
CETP inhibitors	HDL	CETP inhibition	Failed trials	Abandoned	HDL ↑≠ outcome benefit	(Mortensen and Nordestgaard, 2020; Wang et al., 2026)

Lipid metabolism represents a modifiable target for stroke prevention through pharmacologic, lifestyle, and emerging mechanism-based interventions. This table summarizes therapeutic strategies, molecular targets, mechanisms of action, clinical evidence from randomized trials and meta-analyses, regulatory or guideline status, major limitations, and supporting references. Clinical outcomes are presented as relative risk reduction (RRR) where available. Status categories: FDA approved = U.S. Food and Drug Administration–approved for cardiovascular prevention; Guideline-recommended = included in major clinical guidelines; Investigational = preclinical or early-phase clinical development. RRR, relative risk reduction; FDA, Food and Drug Administration; PPARα, peroxisome proliferator-activated receptor alpha; VLDL, very low-density lipoprotein; EPA, eicosapentaenoic acid; DHA, docosahexaenoic acid; AA, arachidonic acid; CETP, cholesteryl ester transfer protein; RCT, randomized controlled trial; LD, lipid droplet; PUFA, polyunsaturated fatty acids.

### 8.3 Emerging therapeutics: mechanism-based approaches

Autophagy enhancers, including rapamycin analogs and spermidine, show preclinical promise in animal models (Aman et al., 2021; Betz and Hall, 2013) but have not been tested in stroke prevention trials. Nicotinamide riboside and NMN restore NAD+ levels in aging animal models (Nguyen et al., 2023); human data for stroke outcomes are not available. Mitochondrial-targeted antioxidants, including MitoQ and SS-31, reduce lipid peroxidation in animal models (Nguyen et al., 2023; Rossman et al., 2018), and MitoQ has shown benefit on vascular function in a small human trial (Rossman et al., 2018), though stroke prevention data are lacking. MAM stabilizers preventing VAPB-PTPIP51 disruption are in early preclinical development (Arjona et al., 2023). Selective ER stress inhibitors targeting PERK and IRE1alpha are in early development; their role in stroke prevention is entirely preclinical (Bhattarai et al., 2021; Hetz et al., 2020). BBB stabilizers, including activated protein C and FGF21, are being explored in experimental models (Andreone et al., 2017; Sweeney et al., 2019). Senolytics, including dasatinib plus quercetin and fisetin, selectively eliminate senescent cells in animal models (Di Micco et al., 2021; Khosla et al., 2020). All these emerging approaches lack randomized clinical trial evidence for stroke prevention and should be framed as investigational.

## 9 Precision prevention: lipidomic risk stratification

Several quantitative lipid-stroke risk estimates have been reported across studies, though effect sizes vary by lipid type and stroke subtype (Gong et al., 2022). Plasma ceramides C16:0, C18:0, and C24:1 have been associated with cardiovascular events independent of traditional markers in prospective studies (Arbaizar-Rovirosa et al., 2023; Shoghli et al., 2025; Vance, 2020; Wei et al., 2024; Yang et al., 2020). Omega-3 index below 4 percent has been associated with increased cardiovascular risk; above 8 percent has been associated with lower risk (Hu et al., 2019; Nayda et al., 2023; Yang et al., 2020). OxLDL/HDL ratio has been proposed to outperform LDL alone in some studies. Lipoprotein particle number, including apoB and LDL-P, has been shown to predict cardiovascular risk better than concentration-based measures in prospective cohorts (Blaha and DeFilippis, 2021). Integrating lipidomic panels with genetic risk, including APOE and PCSK9 variants, and environmental exposure profiles represents a precision medicine hypothesis that has not yet been prospectively validated for stroke-specific outcomes (GBD 2019 Stroke Collaborators, 2021; Vance, 2020; Xu et al., 2025).

Advances in mass spectrometry-based lipidomics now permit comprehensive profiling of hundreds of lipid species from small blood samples, offering unprecedented opportunities for precision stroke risk assessment (Montaner et al., 2020). Table 3 presents lipid biomarkers with established or emerging evidence for stroke risk prediction, ranging from traditional lipid panel components to novel species identified through unbiased metabolomics. The evidence level for each biomarker is indicated; most novel lipidomic biomarkers require further validation in prospective stroke cohorts before clinical implementation.

**TABLE 3:** Lipid biomarkers as stroke risk predictors.

Biomarker	Stroke-associated change	Predictive value (OR or HR)	Study population	Clinical utility	References
LDL cholesterol	↑ ( > 130 mg/dL)	HR ∼1.2–1.5 per 40 mg/dL increase	General population cohorts	Established biomarker and primary therapeutic target (statins, PCSK9 inhibitors)	(Jackler and Ramamurthi, 2019; Mortensen and Nordestgaard, 2020; Wang et al., 2026)
HDL cholesterol	↓ ( < 40 mg/dL in men; < 50 mg/dL in women)	OR ∼0.7–0.8 per 10 mg/dL increase	General population cohorts	Moderate predictive value; limited effective HDL-raising therapies	(Mortensen and Nordestgaard, 2020; Wang et al., 2026)
Triglycerides	↑ ( > 150 mg/dL)	HR ∼1.1–1.3 after LDL adjustment	General population cohorts	Emerging biomarker: therapeutic targeting remains debated	(Khoshnam et al., 2017; Wang et al., 2026)
Lipoprotein(a)	↑ ( > 50 mg/dL)	HR ∼1.4–1.6 in highest quartile	Predominantly European ancestry cohorts	Emerging risk marker; targeted therapies under development	(Jackler and Ramamurthi, 2019; Wang et al., 2026)
Oxidized LDL	↑ Circulating levels	OR ∼1.5–2.0 in highest tertile	Small research and mechanistic cohorts	Investigational biomarker	(Khoshnam et al., 2017; Kumar et al., 2025)
Ceramides (e.g., C16:0, C18:0, C24:1)	↑ Plasma concentrations	HR ∼1.3–1.9 (species-dependent)	Finnish and US metabolomics cohorts	Investigational; promising emerging biomarker	(Khoshnam et al., 2017; Yang Q. et al., 2025b)
Sphingomyelins	Altered circulating species ratios	OR ∼1.2–1.5	Lipidomics-based population cohorts	Investigational	(Khoshnam et al., 2017; Yang Q. et al., 2025b)
Phosphatidylcholines	Altered species composition	Variable, species-dependent associations	Lipidomics cohorts	Investigational	(Khoshnam et al., 2017; Yang Q. et al., 2025b)
Omega-3 Index (EPA + DHA in RBC membranes)	↓ ( < 4% of total RBC fatty acids)	HR ∼0.8–0.9 per 1% increase	Framingham and related prospective cohorts	Emerging biomarker; supplementation trials ongoing	(Lee et al., 2008; Rambold et al., 2015)
Omega-6/Omega-3 ratio	↑ ( > 10:1)	OR ∼1.3–1.7 in highest quartile	US and European cohorts	Investigational biomarker and dietary intervention target	(Lee et al., 2008; Rambold et al., 2015)
Arachidonic acid	↑ Plasma levels	Inconsistent associations	Mixed population cohorts	Unclear clinical utility; conflicting data	(Khoshnam et al., 2017; Lee et al., 2008)
Total cholesterol/HDL ratio	↑ ( > 5)	HR ∼1.5–2.0	General population cohorts	Moderate predictive utility as composite lipid risk marker	(Kealy et al., 2020; Wang et al., 2026)
Non-HDL cholesterol	↑ (Total Cholesterol - HDL)	HR ∼1.3–1.6 per 40 mg/dL increase	General population cohorts	Emerging secondary treatment target	(Kealy et al., 2020; Wang et al., 2026)
ApoB/ApoA1 ratio	↑ ( > 0.9)	HR ∼1.4–1.8	Multi-ethnic population cohorts	Moderate clinical utility; may outperform LDL alone	(Kealy et al., 2020; Wang et al., 2026)
Remnant cholesterol	↑ (Total Cholesterol - LDL - HDL)	HR ∼1.5–2.5 in highest tertile	Danish and US cohorts	Emerging independent risk biomarker	(Kealy et al., 2020; Wang et al., 2026)

Advances in lipidomics enable quantification of lipid species associated with stroke risk. This table summarizes established and emerging lipid biomarkers, their directional associations with stroke risk, effect sizes from major cohort studies, populations studied, current clinical utility, and key references. Predictive values are presented as odds ratios (OR) or hazard ratios (HR) with 95% confidence intervals where available. Clinical utility categories: Established = routinely measured with guideline-supported treatment strategies; Emerging = validated in cohorts but not standard clinical practice; Investigational = requires further validation. OR, odds ratio; HR, hazard ratio; EPA, eicosapentaenoic acid; DHA, docosahexaenoic acid; RBC, red blood cell; ApoB, apolipoprotein B; ApoA1, apolipoprotein A1; TC, total cholesterol; LDL, low-density lipoprotein; HDL, high-density lipoprotein. cholesterol.

## 10 Conclusion and future directions

Environmental factors, including aging, infections, lifestyle, toxins, and stress, have been associated with lipid dysregulation as a plausible common mechanism elevating stroke risk (GBD 2019 Stroke Collaborators, 2021; GBD 2021 Stroke Risk Factor Collaborators, 2024; Wang Y. et al., 2025). The central argument of this review is that environmental exposures converge on lipid metabolic pathways through shared cellular mechanisms, creating cumulative metabolic vulnerability. However, the causal relationships between specific environmental exposures, lipid dysregulation, and stroke outcomes are established to varying degrees, ranging from strong clinical trial evidence for smoking cessation and statin therapy to mechanistic hypotheses that require prospective human validation for most of the cellular pathways discussed.

Key knowledge gaps remain. Longitudinal studies tracking lipid profiles from midlife through stroke events are needed to define critical windows for intervention (Montaner et al., 2020; Vance, 2020). Human postmortem studies of stroke brains rarely include detailed lipidomic analyses, preventing definitive confirmation that experimental findings translate to human disease (Yang et al., 2020). Genetic studies have identified stroke risk loci but rarely mechanistically link them to specific lipid pathways. Clinical trials testing lipid-modifying therapies for stroke prevention have mostly targeted traditional lipids rather than mechanism-based targets such as MAMs, autophagy, ER stress, and specific ceramides.

Future directions should prioritize: first, large prospective cohorts with serial lipidomic profiling to identify predictive signatures (GBD 2019 Stroke Collaborators, 2021; GBD 2021 Stroke Risk Factor Collaborators, 2024); second, Mendelian randomization studies connecting genetic variants to lipid species and stroke to establish causal inference; third, clinical trials of mechanism-based therapies including PPAR agonists, autophagy enhancers, NAD+ boosters, and senolytics in high-risk populations (Di Micco et al., 2021; Khosla et al., 2020); fourth, environmental intervention studies including pollution reduction, dietary changes, and exercise programs with lipidomic endpoints (Feigin et al., 2025; GBD 2019 Stroke Collaborators, 2021); and fifth, precision medicine approaches integrating genomics, lipidomics, exposome data, and clinical factors (Vance, 2020). The ceramide panel (C16:0, C18:0, C24:1) currently represents the lipidomic biomarker closest to clinical implementation and deserves prioritization in prospective stroke prevention trials.

The recognition that environmental exposures may disrupt lipid homeostasis provides a unifying conceptual framework for understanding stroke pathogenesis and opportunities for prevention. By addressing modifiable environmental factors early, targeting lipid metabolism therapeutically where trial evidence exists, and using lipidomic profiling for risk stratification, the field may be able to move toward personalized stroke prevention that reduces the global burden of this devastating disease (Feigin et al., 2025; GBD 2019 Stroke Collaborators, 2021). Realizing this goal will require the prospective human data that this review identifies as the most critical unmet need in the field.
